# FishNet: A dataset of freshwater fish from Bangladesh for deep learning-based fish species classification

**DOI:** 10.1016/j.dib.2026.112799

**Published:** 2026-04-22

**Authors:** Bishal Biswas, Rakibul Haque Rabbi, Md. Mohtashim Masuk, Shah Md Tanvir Siddiquee

**Affiliations:** MARS Lab, Department of Computer Science and Engineering, Daffodil International University, Daffodil Smart City, Birulia, Dhaka 1216, Bangladesh

**Keywords:** Fish identification, Image dataset, Freshwater fish, Fisheries, Computer vision

## Abstract

The fisheries sector plays a vital role in the economy and food security of Bangladesh. Bangladesh is one of the leading countries in inland fish production. Bangladesh gains sustainable economic benefits from aquaculture and fisheries. This sector made a significant contribution to the GDP and ensures employment for approximately 18 million people. Fish is one of the primary sources of protein for the population, accounting for >60 % of the country’s animal protein intake. Efficient fish species identification is relevant to sustainable fisheries management, smart aquaculture, and food authenticity. This dataset includes 2455 clear images of seven frequently consumed freshwater fish in Bangladesh: Shrimp, Prawn, Mola Carplet, Dwarf Gourami, Swamp Barb, Stinging Catfish, and Mystus Catfish. All data were collected from the fish-rich areas in Bangladesh—Netrokona and Bogura. Data samples were collected from ponds, rivers, and fish markets, both natural and commercial sources. The diverse environment provides variation in lighting, background, and orientations, which highlights the real-world complexity for image classification. Each species is classified as scientific, local, and English names for accurate recognition. The dataset is suitable for research in smart aquaculture, including fish identification and species recognition. The collected data allows building machine learning models for image classification and allows fine-tuning previous models for local applications. The dataset includes real-world variability, which may support the generalization and robustness of machine learning models. This dataset provides a strong foundational resource for academic research and practical implementation in smart aquaculture. This dataset aims to contribute to sustainable fisheries and similar ecosystem development.

Specifications Table

**Table:** 

Subject	Computer Sciences
Specific subject area	Image Classification, Image Recognition, object detection, fish species recognition, smart aquaculture.
Type of data	Image (jpg format).
Data collection	The image collection took place over 35 days between 15 March 2025 and 10 April 2025 in Kalmakanda, Netrokona, and Brindabonpara, Bogura. Fish in the dataset are classified into the seven types, which include Shrimp, Prawn, Pabda, Mola, Tengra, Asian stinging catfish, and Puti. The collection of images was captured with two iPhone 11 Pro devices across different camera resolution settings. Images were taken as both JPEG and HEIF formats at resolution settings of 4032×3024 pixels with a 72-dpi & 96-dpi file display quality. Research data was collected from various fish habitats that comprised both natural ponds and commercial markets, as well as flowing rivers. The dataset contains seven separate classes representing each fish species.
Datasource location	Bogura Sadar, Bogura Rajshahi, Bangladesh Coordinates: 24°51′52.4″N 89°22′17.9″E Kalmakanda, Netrokona Mymensingh, Bangladesh Coordinates: 25°04′49.3″N 90°53′24.5″E
Data accessibility	Repository name: Mendeley Data Data identification number: 10.17632/p3xh4fs7cp.3 Direct URL to data: https://data.mendeley.com/datasets/p3xh4fs7cp/3 Instructions for accessing these data: The dataset is publicly available for download at https://data.mendeley.com/datasets/nvhhm5v43x/3
Related research article	None

## 1 Value of the Data

• The dataset contains high-resolution (4032×3024 pixels) images of seven highly consumed fish species in Bangladesh, which makes the dataset an excellent source to design machine learning models and deep learning models for performing fish classification tasks. Deep learning models are highly dependent on the consistency, accuracy, and completeness of the data [1].
• The images were collected from natural and commercial situations (ponds, rivers, and fish markets), which is a rather broad scale of actual life situations. This diversity is what makes the dataset useful in training strong models capable of working with various lighting, background, and pose variations.
• Importantly, this dataset addresses a critical deficiency in publicly accessible resources by providing high-quality images for economically and nutritionally significant Bangladeshi freshwater species. All included species are unique compared to previously published datasets (e.g., Das et al. [2]), highlighting their value for region-specific fish classification research.
• The dataset is useful for studies in smart aquaculture, fish sorting, and recognition, thus making fishery management practices more efficient and sustainable.
• Researchers interested in smart aquaculture, automated species detection, food authentication, and fisheries monitoring may reuse the dataset. It also serves as a benchmark for transfer learning and fine-tuning pre-trained models on the region-specific classification of fish. The dataset can be applied to academic and educational uses to train students in image processing, AI, and agricultural technology, with real-world data on a working basis.

## 2 Background

Fish play an important role in the agriculture, nutrition, and economy of Bangladesh. Millions of people in the country are dependent on fisheries for food and livelihood. Some of the widely consumed fish, such as Freshwater Prawn, Shrimp, Mola Carplet, Dwarf Gourami, Swamp Barb, Stinging Catfish, and Mystus Catfish, make significant contributions to local intakes and markets. Proper detection of these fish plays a vital role in various areas such as sustainable fisheries management, monitoring the supply chain in food, and conservation of biodiversity. Traditionally, species identification is time-consuming and impractical for massive-scale implementation.

Over the last few years, the use of artificial intelligence, in particular, image classification with the use of the deep learning technique, has demonstrated positive outcomes in automatically classifying species. The performance of such models greatly relies on the availability of large, diverse, and high-quality datasets. Unfortunately, there is a lack of publicly available image datasets for Bangladeshi fish species that would allow conducting research and development work.

To fill this gap, we created a dataset consisting of high-resolution images of fish commonly found in Bangladesh. The images were captured using smartphone cameras in different environments. This dataset is intended to support research in machine learning, smart aquaculture, and computer vision by providing a solid foundation for tasks such as fish classification, identification, and recognition based on unique regional features.

Table 1 highlights a comparison of our dataset and an existing dataset. The existing dataset has 12 different species of fish. In our dataset, no common species with the existing ones, and all seven are different. Our dataset mainly focuses on the freshwater native fish.

**Table 1:** Comparison of an existing dataset.

Criteria	Proposed Dataset	Existing Dataset (Das et al. [2])
Number of Species	7	12
Total Images	2455	4939
Unique Species	Shrimp, Prawn, Mola Carplet, Dwarf Gourami, Swamp Barb, Stinging Catfish, Mystus Catfish	Black Rohu, Catla, Common Carp, Freshwater Shark, Grass Carp, Long-whiskered Catfish, Mirror Carp, Mrigal, Nile Tilapia, Silver Carp, Striped Catfish
Common Species	None	None
Main Contribution	Focus on local fish species	A broader mix of commercially common species
Image Source	Self-collected images	Publicly available

## 3 Data Description

The dataset comprises high-quality images captured in Bangladesh, intended for both image classification and fish identification. Those fish are Freshwater Prawn, Freshwater Shrimp, Mola Carplet, Dwarf Gourami, Swamp Barb, Stinging Catfish, and Mystus Catfish. Images were taken using two iPhone 11 Pro, with dimensions 4032×3024 pixels. There are seven folders containing those images. Images were captured in the period running from March 15 to April 10 from natural habitats, and the fish market in Netrokona and Bogura. This dataset will be useful for image classification, and researchers want to develop an automatic fish identification system.

Table 2 represents the local names, English names, and scientific names of the fish. It also provides the total number of images in the dataset for each class.

**Table 2:** Description of the fish.

SI	Local Name	English Name	Scientific Name	Number of Images
1	Chingri	Shrimp	*Macrobrachium nipponense*	122
2	Chingri	Prawn	*Macrobrachium malcolmsonii*	225
3	Mola	Mola Carplet	*Amblypharyngodon mola*	405
4	Kholisha Fish	Dwarf Gourami	*Trichogaster lalius*	228
5	Puti	Swamp Barb	*Puntius chola*	495
6	Shing	Stinging Catfish	*Heteropneustes fossilis*	549
7	Tengra	Mystus Catfish	*Mystus tengara*	431
			Total:	2455

Table 3 represents the fish description of the dataset and detailed information about the fish. This table highlights the species names, local names, distinguishing features, and typical habitats of each fish included in the dataset.

**Table 3:** Detailed description of fish.

Class	Description	Image
Shrimp	Shrimp (*Macrobrachium nipponense*) are small aquatic invertebrates found in both freshwater and marine environments. Depending on the species, the average length of Shrimp is about 4–8 cm. Between 22–28 °C temperatures with a pure & well-oxygenated environment, Shirm can survive. Shrimp is rich in essential amino acids such as lysine, methionine, and tryptophan [3]. Some species of shrimp are cultivated in farms. (See **Fig. 1**)	**Fig. 1.** Freshwater Shrimp.
Prawn	Prawn (*Macrobrachium malcolmsonii*) are marine or Freshwater creatures that are generally longer than shrimp. For variations of species, it can grow up to a length of 15-25 cm. Prawn belong in warm and pure water with temperatures between 25 and 30 °C. Prawn are generally cultivated in farms for food and are also sold globally in markets. They show rapid growth and can thrive in a wide range of farming conditions [4]. (See **Fig. 2**)	**Fig. 2.** Freshwater Prawn.
Mola Carplet	Mola Carplet (*Amblypharyngodon mola*) is a small freshwater fish. It is commonly found in ponds, rivers, and floodplains of South Asia. This fish is locally known as Mola in Bangladesh. This fish is widely consumed due to its high vitamin A content and high nutritional value [5]. Its slender body and silvery scales help to identify the fish. This fish is important for both ecological research and food security. (See **Fig. 3**)	**Fig. 3.** Mola Carplet.
Dwarf Gourami	The Dwarf Gourami (*Trichogaster lalius*) is a small South Asian fish that belongs in slow-moving water. Generally, it grows up to 6–8 cm long. Commonly, it has iridescent blue and red or orange vertical stripes of color. This fish can survive under 22–28 °C temperature, along with a 6.0–7.5 pH value. This species is very peaceful and shy. Sometimes it gets into community aquariums. It has Omnivorous feeding behavior. Living in acidic or pure(deionized) water, the Dwarf Gourami can change the appearance and activity of its gill arches and kidneys [6]. (See **Fig. 4**)	**Fig. 4.** Dwarf Gourami.
Swamp Barb	The Swamp Barb (*Puntius chola*) lives in low-depth water, like muddy lands and soft wetland places. It is a small native freshwater fish from the Cyprinidae family, valued for both food and ornamental purposes, and is found in Pakistan, India, Nepal, Bangladesh, Myanmar, and Sri Lanka [7]. It is identified by its Light red-colored fins, thick body structure, and dark side stripe along its side. For larger predators, and contributes to nutrient cycling in Aquatic habitats, it serves as a significant forage fish. (See **Fig. 5**)	**Fig. 5.** Swamp Barb.
Stinging Catfish	The Stinging Catfish (*Heteropneustes fossilis*)*,* which stays in clean water, saltish water environments, and in tranquil waters in South Asia. It can also survive in dirty water with low oxygen. It has sharp spines that can sting, meaning it can inject venom which can cause pain and swelling, a long and thick body shape, dark brown color. On average, the growth of its 20–30 cm in length and an average weight of 100–300 g Due to the lack of proper cultivation techniques, the culture of H. fossilis has not yet developed well in Bangladesh [8]. (See **Fig. 6**)	**Fig. 6.** Stinging Catfish.
Mystus Catfish	The Mystus Catfish (*Mystus tengara*) is a tiny freshwater fish that lives in ponds, rivers, and canals in South Asia. Mystus Catfish belongs to the Bagridae family. Usually it is about 10–15 cm long and average weight of around 50–150 g Generally, the fish is identified by a special mix of features: a clear spot near the ear area, four brown stripes on its body with narrow pale gaps in between, and its small fin on the back starts before the last back fin ray [9]. It is active at night. (See **Fig. 7**)	**Fig. 7.** Mystus Catfish.

### 3.1 Significance of the dataset

Fish are one of the most important sources of high-quality protein and micronutrients essential for human health [10]. Automatic identification of fish using machine learning and computer vision is very popular at present. Data quality is a crucial factor in computer vision to get the desired output of an algorithm [11]. Researchers are classifying fish species using deep learning, as it is an attractive and popular domain.

This dataset has a significant value in the development of new digital solutions for the fisheries sector in Bangladesh. This dataset provides high-quality images of seven freshwater fish. Images were captured in both natural and commercial environments. This dataset will address the gap in publicly unavailable data on Bangladeshi native fish. This dataset will help to develop automated fish identification, monitor biodiversity, and management of fish resources. Notably, Mola (*Amblypharyngodon mola),* included in the dataset, is a great source of vitamin A, highlighting its nutritional significance in prior studies [5].

Having diverse and regularly available visual material allows the dataset to help in many projects, including education, protection of aquatic life, organizing stock, and inventing new ways to identify fish using IT systems. Additionally, it allows for projects that study smart aquaculture in the local area, leading to inventions fitting the needs of that region.

Table 4 represents a brief description of the dataset that contains the images. It provides information about the image format, size, processed image information, and the total images in the dataset.

**Table 4:** Brief description of the dataset.

No.	Particular	Description
1	Fish	Shrimp, Prawn, Mola Carplet, Dwarf Gourami, Swamp Barb, Stinging Catfish, and Mystus Catfish
2	Original Image	HEIF, JPEG; *4032×3024 pixels; 72 dpi and 96 dpi.*
3	Processed Image	JPG; 640×640 pixels
4	Annotation	None
5	Dataset size	Original image folder size: 3.15 GB Size of each image: 0.6 MB – 2.5 MB Processed Image folder size: 276 MB Size of each image: 60 K – 200 kb Total Size: 3.4 GB
6	No. Of subfolders	7

### 3.2 Dataset folder description

**Fig. 8:**
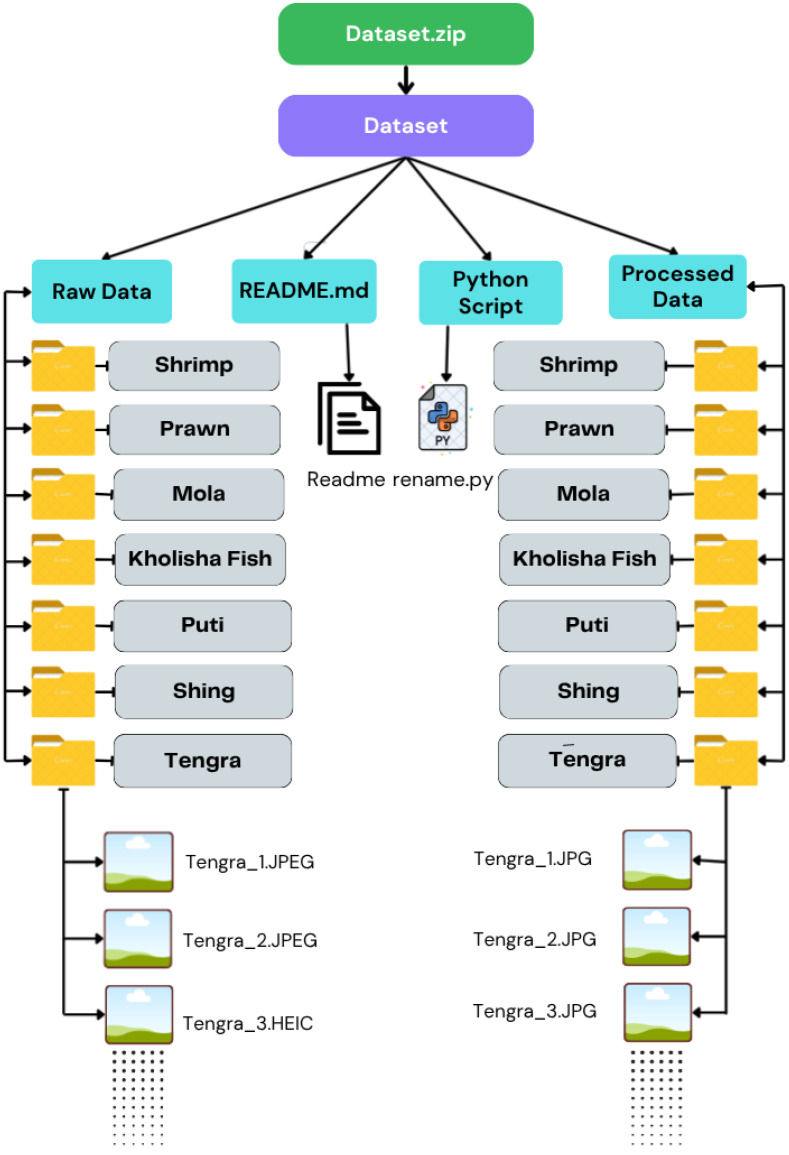
Description of the dataset folder.

This dataset folder is named “Dataset.zip” as the root file is a zip format. The root file contains a Dataset file, and that file contains 7 subfolders. Each subfolder contains corresponding images of the dataset. Each image of the dataset is renamed with the folder name starting from 1. The folder and images were renamed with the local name. The visual representation of the dataset is in Fig. 8.

## 4 Experimental Design, Materials, and Methods

### 4.1 Fieldwork and theoretical knowledge

Theoretical knowledge provides a strong foundation of understanding about core concepts, principles, and methodologies relevant to a field. Proper theoretical knowledge and fieldwork are required to collect a proper dataset. Before collecting the data, we conducted a study to learn about the fish in freshwater. We visited various fish markets and environmental sites to collect data.

### 4.2 Digital image acquisition

Digital image acquisition refers to the process of capturing data. Data quality plays a significant role in computer vision and deep learning. Clear and high-quality images have an impact on the result of the deep learning models.

• The camera angle ranged between 45° to 90°, depending on the position of the fish. As fish were in different environments, camera angle and settings were adjusted as required to capture the features of the fish, like body, shape, color patterns, and distinguishing marks.
• Images were captured at a distance of 30 cm to 60 cm from the fish. To ensure the image quality and image clarity, images were captured from appropriate vantage points. This optimal distance ensured that sharp, detailed images were captured without distortion.
• Each fish was placed on a white board during photography to provide a uniform background, reduce visual distractions, and ensure clear visibility of distinguishing features such as body shape, fins, and color patterns.
• The images were collected from multiple freshwater fish sources in Bangladesh during the year 2025. Specifically, data were obtained from Bogura Sadar, Bogura, Rajshahi (Coordinates: 24°51′52.4″N, 89°22′17.9″E) and Kalmakanda, Netrokona, Mymensingh (Coordinates: 25°04′49.3″N, 90°53′24.5"E). These locations represent a mix of natural and commercial environments, including ponds, rivers, and local fish markets, ensuring that the dataset reflects real-world conditions for the captured species.

**Fig. 9:**
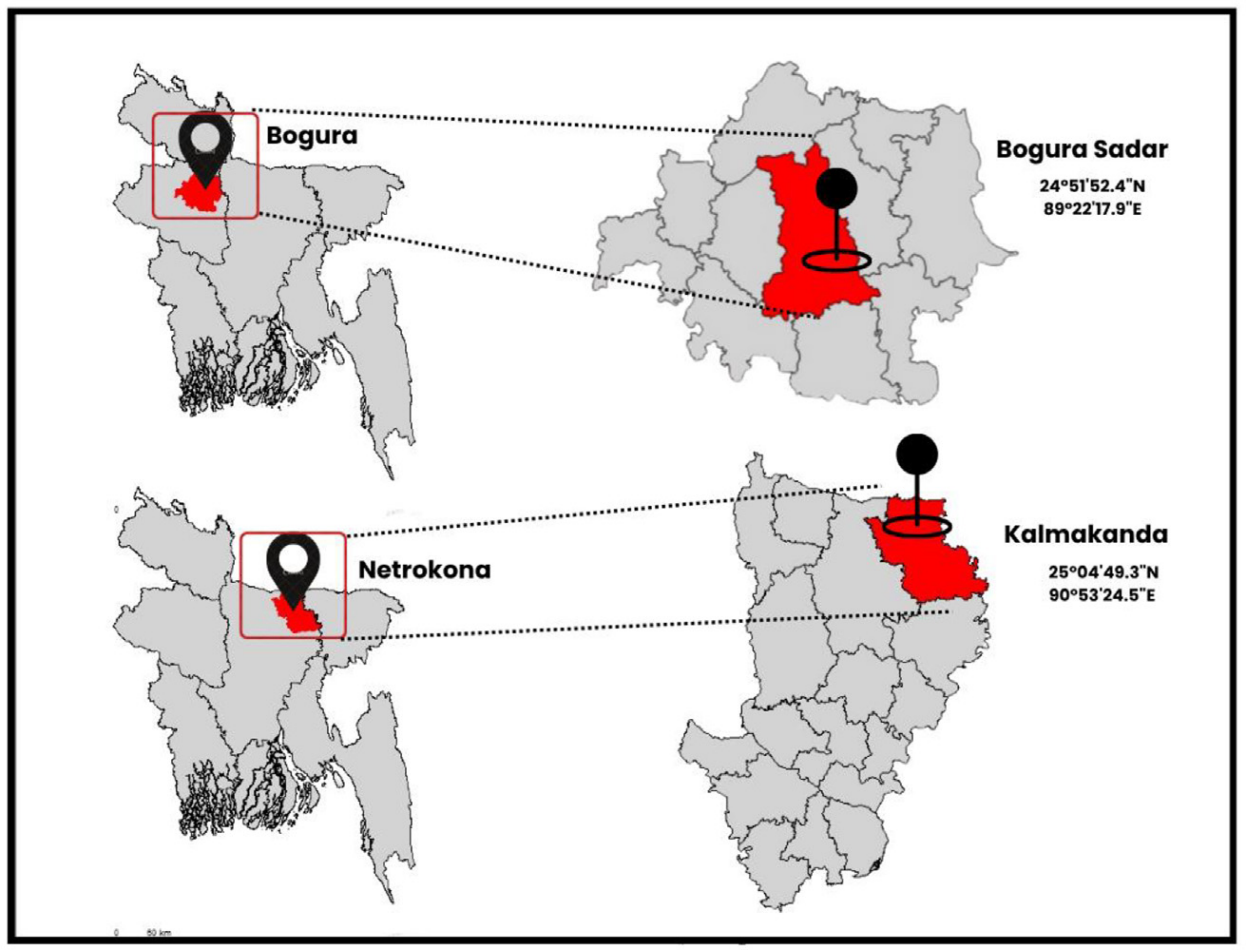
Data collection area.

Fig. 9 shows the geographical location of data collection areas. Data were collected from Bogura and Netrokona. In the map, data collection areas were pinpointed.

The images were captured in a high resolution (4032×3032 Pixels) with two Apple iPhone 11 Pro, stored in 24-bit color depth. The two devices were positioned differently during image taking, although they were of the same model, to obtain a range of image characteristics. The devices support HEIF and JPEG formats, with HEIF being the default high-efficiency option. HEIF (High Efficiency Image Format) is a modern image container that can store single images, while HEIC (High Efficiency Image Coding) is a specific type of HEIF that uses HEVC compression, producing smaller file sizes without compromising image quality. In this dataset, one device captured images in HEIC format, and the other supported both HEIF and JPEG, depending on the camera settings, ensuring compatibility and high-quality image acquisition.

Table 5 represents the device information used for image acquisition. Two devices are used to capture the data.

**Table 5:** Description of the devices used in image acquisition.

Particulars	Device Information	
Device name	iPhone	iPhone
Manufacturer	Apple	Apple
Model	11 Pro	11 Pro
Camera pixel	12 MP, 12 MP, 12 MP	12 MP, 12 MP, 12 MP
Aperture Value	f/1.8	f/1.8
Exposure time	1/122 s	1/127 s
Camera flash	No flash	No flash
Monitoring range	4032×3024	4032×3024
Image Metadata	72 /96 dpi Bit Depth 24	72/96 dpi Bit Depth 24
Image format	HEIF/JPEG	HEIF

Images were captured with a similar device, but due to different camera settings, capturing environments, automatic adjustments in exposure, focus, white balance, and HDR processing, image metadata may vary. DPI (dots per inch) may vary from image to image, even when captured on the same device, because DPI is a metadata property rather than a fixed physical attribute of the sensor. In general, using two devices that had slightly different settings increased the diversity of the data, and high-quality images can be applied in computer vision, deep learning, and fish species classification research.

### 4.3 Image classification and processing

Image Classification and processing are essential and crucial steps in preparing a dataset for computer vision and deep learning applications. Once the images were acquired, each image underwent preprocessing. After the image acquisition, the data were curated properly to ensure the dataset quality.

#### 4.3.1 Data curation

All the captured images underwent a thorough quality assessment to ensure the dataset quality, integrity, and reliability. After image acquisition, all the low-quality images were removed from the dataset. Images with low detail, blurriness, poor lighting, and partially captured were not included in the dataset for better consistency. Properly capturing the images ensures creating a quality dataset.

#### 4.3.2 Image classification criteria

After data curation, all images were systematically classified according to fish species to ensure accurate labeling and dataset consistency. The classification protocol involved the following steps:

• Initial Labeling: The images were initially labeled using prior knowledge, literature review, related journal references, and expert opinions from online resources. This preliminary labeling was further guided and validated by a fisheries specialist with an academic background in fisheries and aquaculture, ensuring accurate identification of fish species.
• Expert Review: A fisheries specialist reviewed all images to confirm species identification, using distinguishing features such as body shape, fin structure, color patterns, and scale arrangement, ensuring accurate and reliable final classification.
• Final Categorization: Validated images were organized into a structured directory for each species, ready for training, testing, and evaluation in deep learning models.

**Fig. 10:**
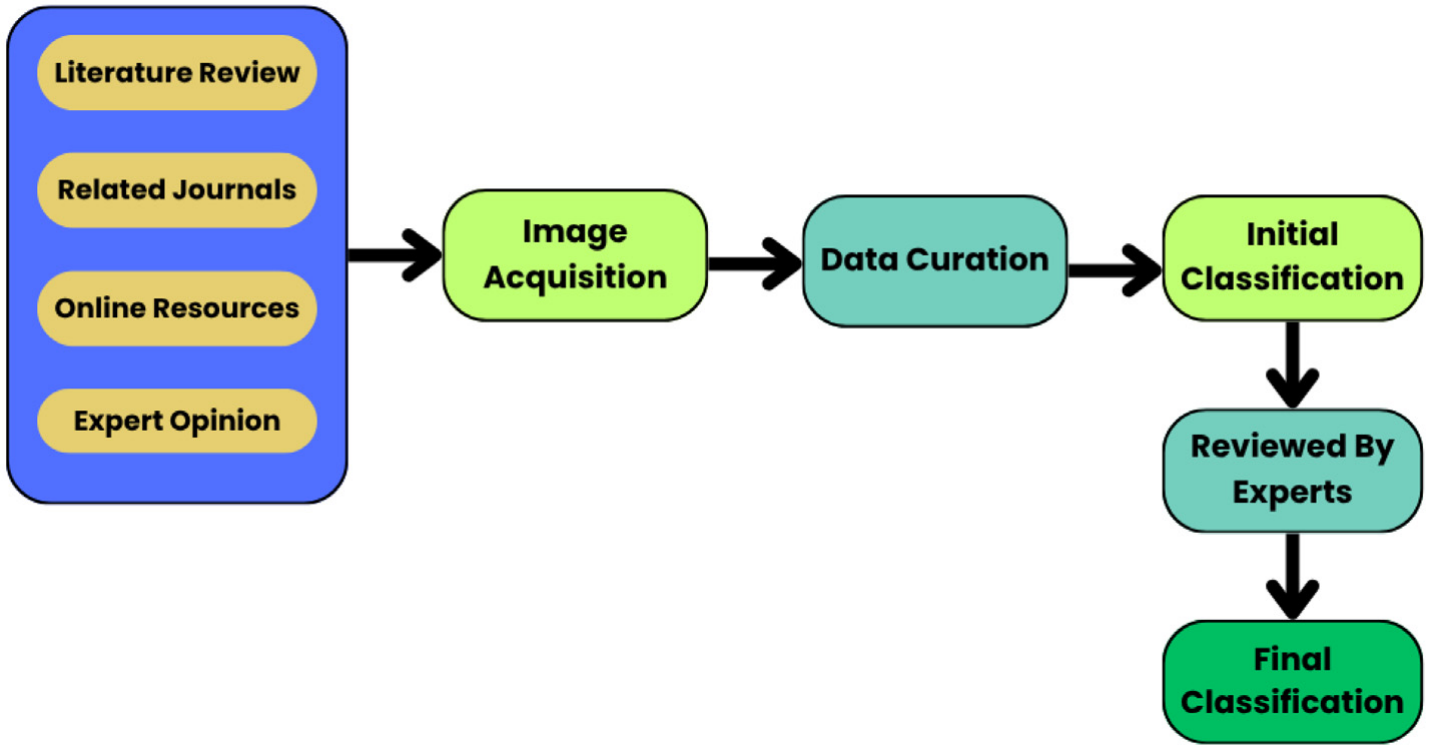
Classification criteria of the dataset.

This protocol ensures that the dataset is accurately labeled, reproducible, and suitable for robust computer vision analyses (see Fig. 10).

The raw images in the dataset are originally in high resolution (4032×3024 pixels). To optimize the dataset size and make it accessible to researchers, the images were reprocessed by resizing them to 640×640 pixels using a Python script. This resizing was performed exclusively in Google Colab to maintain consistency across the dataset. Additionally, the images were renamed according to their respective folder names using Python script. A single Python script was used to resize, rename, and convert the image formats. The Python script used for preprocessing is provided to ensure repeatability and transparency of the dataset preparation. The Python scripts are included in the data repository within a dedicated folder, and include a README.md file describing the dataset organization for better accessibility

Though shrimp and prawn are not taxonomically identified as fish, we added them to the dataset due to their ecological significance in the freshwater environment, as well as their common culinary and commercial value in Bangladesh. Incorporation of these species, in addition to freshwater fish, will mean that the set of data will be representative of the richness of aquatic species most likely to be consumed in the area, and will be a better source of information on aquaculture, fisheries monitoring, and species classification.

## Limitations

This dataset doesn’t cover all the fish species found in freshwater. Shrimp and prawn also have less data. There are enormous differences between shrimp, prawn, and other species. The data were collected from only two locations in Bangladesh, which is a geographical limitation for the dataset. This dataset is not annotated by any expert. Commercial environment image capturing was a challenge. We have included two species of shrimp and prawn under the category of fish in our study, where shrimp and prawn are not classified as fish. This is because, in Bangladesh and many other South Asian countries, these species are ecologically cultivated and considered fish.

## Ethics Statement

The authors have read and obeyed the ethical requirements for publication in Data in Brief and confirm that the data collection involved photographing live fish from a pond without causing harm or invasive procedures.

## CRediT authorship contribution statement

**Bishal Biswas:** Writing – original draft. **Rakibul Haque Rabbi:** Writing – original draft, Visualization. **Md. Mohtashim Masuk:** Writing – review & editing. **Shah Md Tanvir Siddiquee:** Supervision, Writing – review & editing.

## Data Availability

Mendeley DataFishNet: A High-Resolution Image Dataset for Automated Fish Species Recognition (Original data).
